# Key evidence of the role of desertification in protecting the underlying permafrost in the Qinghai–Tibet Plateau

**DOI:** 10.1038/srep15152

**Published:** 2015-10-15

**Authors:** Shengbo Xie, Jianjun Qu, Yuanming Lai, Xiangtian Xu, Yingjun Pang

**Affiliations:** 1 Key Laboratory of Desert and Desertification/State Key Laboratory of Frozen Soil Engineering/Dunhuang Gobi and Desert Research Station, Cold and Arid Regions Environmental and Engineering Research Institute, Chinese Academy of Sciences, Lanzhou 730000, China; 2 Institute of Transportation, Inner Mongolia University, Hohhot 010070, China; 3 Institute of Desertification Studies, Chinese Academy of Forestry, Beijing 100091, China

## Abstract

Previous research has shown that the temperature of underlying permafrost decreases after the ground surface is covered with sand. No significant conclusions have yet been drawn that explain why this happens, because the heat transfer mechanism effects of the sand layer on the underlying permafrost remain unclear. These mechanisms were studied in the present work. We found that the upward shortwave radiation flux of the Qinghai-Tibet Plateau ground surface with a sand layer covering was higher than that of the surface without sand; thus, the atmospheric heat reflected by the sand layer is greater than that reflected by the surface without sand. Therefore, the net radiation of the surface with the sand layer is lower than that of the surface without sand, which reduces the heat available to warm the sand layer. Because sand is both a porous medium and a weak pervious conductor with poor heat conductivity, less heat is conducted through the sand layer to the underground permafrost than in soil without the sand deposition layer. This phenomenon results in a decrease in the ground temperature of the permafrost under the sand layer, which plays a key role in protecting the permafrost.

The Qinghai–Tibet Plateau features a permafrost region with a total area of 149 × 10^4^ km^2^. This region is the world’s largest high–altitude permafrost area in the middle and low–latitude zones and accounts for 69.3% of the total permafrost area in China. This unique environment, which is characterized by high elevation and low temperature^1^,^2^,^3^, is distinguished by frequent freeze–thaw cycles^4^, strong frost weathering and abundant sources of sandy materials. Under dry and windy climate conditions, release and elutriation of sandy materials on the ground surface increase, the wind–sand activity is strong^5^,^6^,^7^,^8^,^9^, and desertification is frequent^10^. These natural processes result in various sand depositions on the ground surface, which result in changes to the properties of the Qinghai–Tibet Plateau ground surface. Such changes, in turn, affect the radiation and energy balance at the ground surface^11^ as well as water and heat fluxes in the ground^12^,^13^,^14^,^15^. In the permafrost regions of the Plateau, the ecological environment, particularly the permafrost environment, is highly fragile^16^,^17^; even a minimal change in the ground surface properties can influence the underlying permafrost through various processes, including radiation, water and heat exchange^18^.

Several recent studies have been conducted on variation in the underlying permafrost after the ground surface is covered with sand, including the role of a sand layer on the convective cooling effect often observed in permafrost areas^19^,^20^. An interesting finding at the Honglianghe River of the Qinghai–Tibet Plateau is that permafrost temperatures underneath the sand layer are lower than that of permafrost underneath surfaces without sand cover (Fig. 1)^21^. This result has caused great concern. Thus, it is important to determine why permafrost temperatures decrease under sand layers. The reasons behind this decrease in temperature are currently unclear. The potential influence of future climate warming on permafrost in cold regions provides the impetus for seeking a better understanding of the influence of sand layers on underlying permafrost^22^,^23^. Therefore, the authors conducted an experiment on the Qinghai–Tibet Plateau hinterland for field observation, combined with laboratory analyses and tests, to investigate the heat transfer mechanisms of the ground surface sand layer on the underlying permafrost in this region.

## Experimental design and research methods

**Figure 2:**
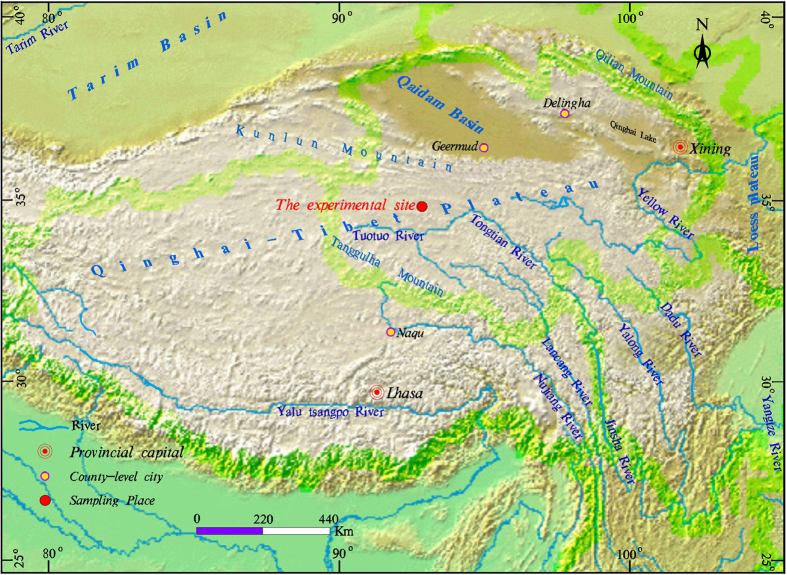
Location map of Honglianghe River of the Qinghai-Tibet Plateau (the map was edited and generated by Mapgis 6.7 which is a Chinese GIS software, Shengbo Xie created this map).

**Figure 3:**
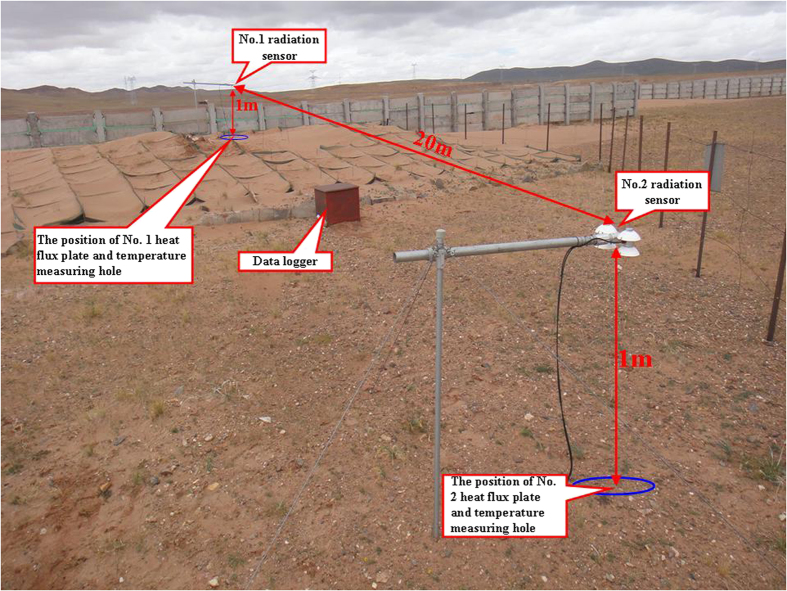
Positions of the observation points at the experimental field (the photograph taken by Shengbo Xie at the experimental field of Honglianghe River of the Qinghai–Tibet Plateau).

Research data were obtained by field observation and laboratory experiments. Variations in radiation and reflectance on the surface of the sand layer, the heat flux in the sand layer, and the temperature of the permafrost underneath the sand layer were observed by means of the synchronization contrast method^21^. The site for the observational study was located at the Honglianghe River of the Qinghai–Tibet Plateau. The geographic coordinates of the study area are 35°03’13” N, 93°01’07” E, and the plateau has an altitude of 4,658 m. The study site was located on the Qinghai–Tibet Plateau hinterland (Fig. 2), where the vegetation is sparse, the dunes are widespread on the ground surface, and permafrost has developed underground. The convective cooling effect is not obvious at this study site due to a lack of coarse blocky material. We selected a typical dune covering an area of 20 m × 15 m that had an average sand bed thickness of 1 m. The dune centre is the primary observation area. A net radiometer with four components at a height of 1 m above the sand surface was erected on the sand layer (Hukseflux Thermal Sensors, The Netherlands). A heat flux plate was buried at a depth of 0.05 m below the surface of the sand layer (Hukseflux Thermal Sensors). A temperature–measuring hole was dug underneath the sand layer to observe the ground temperature of the permafrost. In addition to the dune, a natural ground surface without sand deposition was selected as the second observation area. The soil texture of the ground surface without sand deposition was that of argillaceous siltstone. A net radiometer with four components at a height of 1 m above the surface was also erected on this surface, and a heat flux plate was buried at a depth of 0.05 m below the surface of the soil. A temperature–measuring hole was dug underneath the natural ground surface to observe the ground temperature of the permafrost. The distance between the first and second observation areas was 20 m (Fig. 3). The instruments were connected by data lines to the automatic data logger. The data logger collected observation data from the net radiometers and heat flux plates every 30 min, with the total observation time lasting for one year (September 2012 to August 2013). Local sand and soil samples were also collected. The granularity of the samples was tested in the laboratory using a laser particle size analyzer, and thermal conductivity was determined using the unsteady state hot wire method.

## Research results

### Albedo

**Figure 4:**
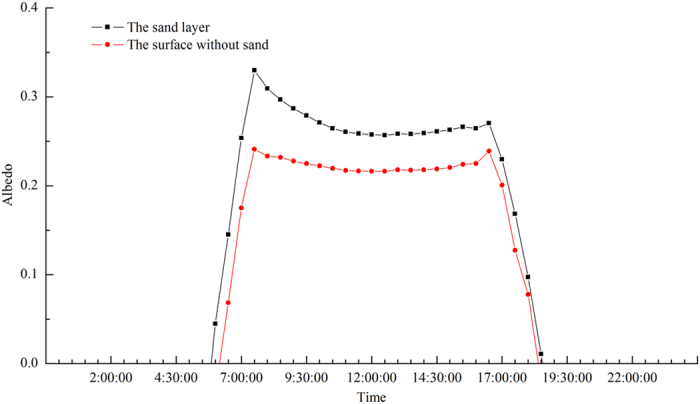
Intra-daily variation of albedo averaged over each day of one year at the Honglianghe River of the Qinghai–Tibet Plateau (September 2012 to August 2013).

The annual average albedo of the sand layer at the Honglianghe River of the Qinghai–Tibet Plateau is 0.30, whereas the annual average albedo of the surface without the sand layer is 0.25. Based on the diurnal variation graph of the annual average albedo (Fig. 4), the albedo of the sand layer began to increase between 5:30 and 6:00, followed by a rapid increase to a maximum of 0.33 at 7:30, which is the maximum albedo for the day. The maximum albedo in the afternoon was 0.27, which was reached at 16:30. Thereafter, the values decreased rapidly and returned to zero between 18:30 and 19:00. The albedo of the surface without the sand layer began to increase from 6:00 to 6:30 and then increased rapidly to a maximum of 0.24 at 7:30. This value was maintained until 16:30; thereafter, the value decreased rapidly and returned to zero between 18:00 and 18:30. In addition to the annual average albedo of the sand layer being higher than that of the surface without sand, the diurnal variation, daily maximum of the annual average albedo, and duration of the positive value of the former were all greater than those of the latter (Fig. 4).

### Radiation

**Figure 5:**
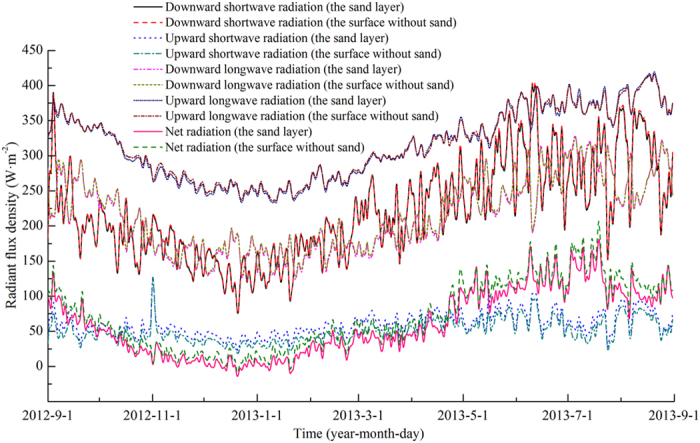
Variation in radiant flux density within the year at the Honglianghe River of the Qinghai–Tibet Plateau.

The four–component radiation and net radiation observations for the two study sites are shown in Table 1 and Fig. 5. For both the sand layer and the surface without sand, the annual average flux density and yearly total are highest for the upward longwave radiation and lowest for the upward shortwave radiation. The flux densities for downward shortwave radiation and downward longwave radiation are intermediate, with minimal differences observed between these last two components. The flux densities of the four-component radiation during summer and autumn are greater than those during winter and spring. For both the annual averages and yearly total values, the flux densities for downward shortwave, downward longwave, and upward longwave radiation for the sand layer are nearly equal to those for the surface without sand. However, flux density for the upward shortwave radiation of the sand layer is significantly greater than those of the surface without sand, with increases of 9.50 W·m^−2^ for the annual averages and 299.81 MJ for the yearly totals. The observed net radiation values during summer and autumn are greater than those during winter and spring. The annual average value of the net radiant flux density and the yearly total value of net radiation of the sand layer are significantly lower than those of the surface without sand, with corresponding values showing decreases of 11.17 W m^−2^ and 352.16 MJ.

**Figure 6:**
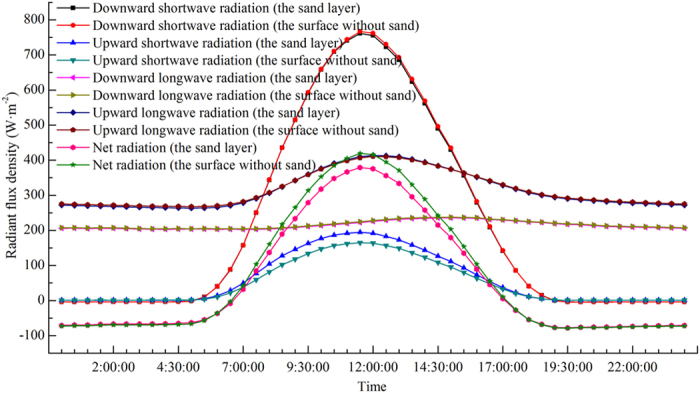
Intra-daily variation of radiant flux density averaged over each day of one year at the Honglianghe River of the Qinghai–Tibet Plateau (September 2012 to August 2013).

The diurnal variation of the annual average radiant flux density is shown in Fig. 6. Based on the four–component radiation observations, the four–component radiant flux density during the day is greater than that during the night, both in the sand layer and in the surface without sand, and reached peak values at noon. The downward shortwave radiation exhibited the maximum diurnal flux density amplitude, followed by, in decreasing order, the upward shortwave radiation, the upward longwave radiation and the downward longwave radiation. The net radiant flux density in both the sand layer and the surface without sand is negative at night, with minimal changes. The net radiant flux density turned from negative to positive between 6:30 and 7:00, increased gradually to a maximum value at 12:00 noon, and then decreased gradually. The net radiant flux density turned from positive to negative between 17:00 and 17:30. The net radiant flux density during the day is greater than that during the night, and the net radiant flux density of the sand layer is nearly equal to that of the surface without sand during the night. However, the net radiant flux density of the sand layer is significantly lower than that of the surface without sand during the day, with a maximum difference of 40.47 W·m^−2^.

### Soil heat flux

**Figure 7:**
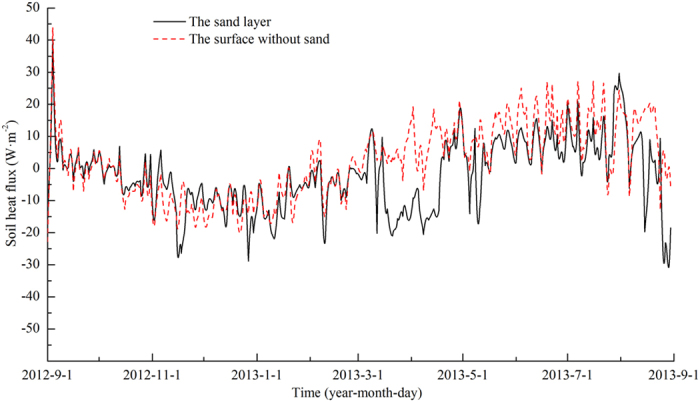
Variation in the soil heat flux within the year at the Honglianghe River of the Qinghai–Tibet Plateau.

**Figure 8:**
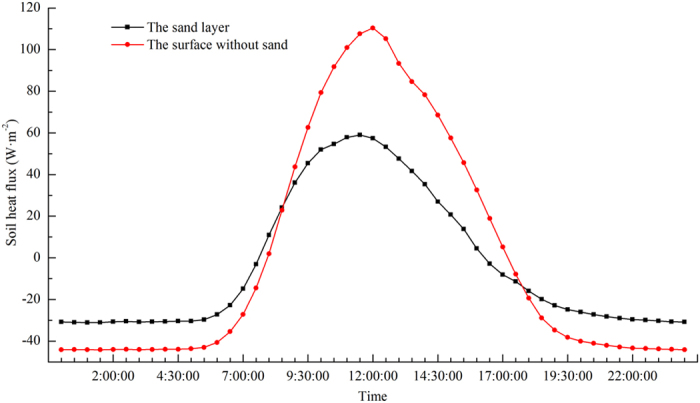
Intra-daily variation of the soil heat flux averaged over each day of one year at the Honglianghe River of the Qinghai–Tibet Plateau (September 2012 to August 2013).

Both the sand layer and the surface without sand at the Honglianghe River of the Qinghai–Tibet Plateau are dominated by heat absorbing during summer, when the direction of soil heat flux is downwards; heat is released during winter, when the direction of soil heat flux is upwards (Fig. 7). Heat is absorbed during the day and released at night (Fig. 8). Diurnally, the soil heat flux of the sand layer increased significantly from 6:00, transitioned from heat release to heat absorbing between 7:30 and 8:00, and reached maximum absorbing at 11:30 (Fig. 7). Thereafter, the soil heat flux decreased and changed from heat absorbing to heat release between 16:00 and 16:30. The soil heat flux did not change significantly during the night. The soil heat flux of the surface without sand increased significantly from 6:00, turned from heat release to heat absorbing between 7:30 and 8:00, and reached maximum absorbing at 12:00 noon. Thereafter, the soil heat flux decreased and changed from heat absorbing to heat release between 17:00 and 17:30. The soil heat flux did not change significantly during the night. The duration of heat absorbing by the sand layer during the day is shorter than that by the surface without sand, and the absorbing is less extensive. The diurnal amplitude and the annual average value of the soil heat flux of the sand layer are lower than those of the surface without sand.

### Analyses and discussion

The upward shortwave radiation observed after the ground surface was covered with sand is greater than that of the surface without sand because the albedo of the former is higher than that of the latter. When the radiation fluxes of other components are nearly equal, the sand layer reflects more atmospheric heat than does the surface without sand. Therefore, the net radiation of the sand layer is lower than that of the surface without the sand layer, which means that less heat is available to warm the surface of the sand layer than the surface without sand. Based on the Stefan–Boltzmann law, we have
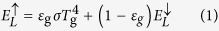


in which 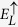 is the upward longwave radiation of the ground surface (W·m^−2^); *ε*_g_ is the emissivity of the ground surface; σ is the Stefan–Boltzmann constant (5.6696 × 10^−8^ W·m^−2^·K^−4^); 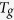 is the temperature of the ground surface (°C); and 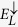 is the downward longwave radiation of the atmosphere (W·m^−2^).

Thus, we have:
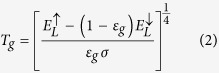


Based on the equation (2), if the emissivity of the ground surface is 0.95, the temperature of the surface of the sand layer must be 1.07 °C and the temperature of the surface without the sand layer must be 1.39 °C. Thus, the temperature on the surface of the sand layer is lower than that of the surface without sand, as observed. If molecular conduction is the main process of soil heat transmission in homogeneous soil, the Fourier law can be used to approximately the heat fluxes at a given depth of the soil layer:
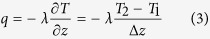


where *λ* is the thermal conductivity of the soil layer (W·m^−1^ °C^−1^). Based on the laboratory test results, the thermal conductivity of the sand and soil sample are 0.36 and 0.43 W·m^−1^ °C^−1^, respectively. Here, *T* is the temperature of the soil layer (°C), *T*_1_ is the temperature value of the upper surface of the soil layer, *T*_2_ is the temperature value of the lower surface of the soil layer, and Δz is the thickness of the soil layer (m).

Based on equation (3), the heat fluxes of the sand layer at depths of 0.5 and 1 m are 0.17 and 0.12 W·m^−2^, respectively. The heat fluxes of the surface without sand at depths of 0.5 and 1 m are 0.76 and 0.46 W·m^−2^, respectively. The heat flux in the sand layer is less than that in the surface without sand. These results are consistent with the heat flux results measured in the field.

Based on analysis of the laboratory samples, the particle sizes for the sand in the experimental field at the Honglianghe River were predominately in the range of 0.10–0.25 mm and 0.25–0.50 mm. Sand with particle sizes in the range of 0.05–0.10 mm was found infrequently, and sand with particle sizes in the range of 0.001–0.005 and 0.005–0.05 mm was not found (Table 2). A significant amount of space was also present between sand particles. The soil sample showed different characteristics. Although particle sizes in the range of 0.10–0.25 mm dominated the study area soil, particle sizes in the ranges of 0.001–0.005 mm, 0.005–0.05 mm, and 0.05–0.10 mm, were also fairly high (Table 2). The gaps between soil particles were mainly filled with fine materials, which caused the porosity of the soil to be less than that of the sand layer.

The thermal conductivity of the sand layer is smaller than that of the soil layer^13^. Under certain atmospheric driving conditions, heat flux within soil is influenced by a number of factors, such as thermal diffusion coefficient and water content^12^,^15^. Compared with the soil without the sand deposition layer, the sand layer has a lower water content and a better separation of sandy material (Table 2). These characteristics give the sand layer a low soil heat flux and low ground temperature amplitude^14^. The sand layer is clearly both a porous medium and a weak pervious conductor with poor heat conductivity; therefore, the heat conducted through the sand layer to the underground permafrost is less than that in the soil without the sand layer.

Given the rapid developments in engineering construction projects that are undertaken in cold regions^24^,^25^,^26^,^27^, preventing permafrost degradation is a primary concern of academic communities^28^,^29^,^30^,^31^,^32^. The decrease in ground temperature of the permafrost under a sand layer was confirmed by the experimental results and analyses of this study. These results present guiding significance for protecting the permafrost in sandy regions of the Qinghai–Tibet Plateau and other similar zones where engineering construction projects are required.

## Conclusions

The upward shortwave radiation flux of the ground surface of the Qinghai–Tibet Plateau covered with a sand layer increases because the surface albedo increases. When the radiation fluxes of other components are nearly equal, the atmospheric heat reflected by the sand layer increases. Therefore, the net radiation of the sand layer decreases, which reduces the heat available to warm the surface of the sand layer.

The soil heat flux decreases after the ground surface of the Qinghai–Tibet Plateau is covered with sandy depositions because the sand layer is both a porous medium and a weak pervious conductor with poor heat conductivity, therefore, the heat conducted through the sand layer to the underground permafrost decreases. This phenomenon induces the ground temperature of the permafrost to decrease under the sand layer, which plays a key role in protecting the permafrost.

## Additional Information

**How to cite this article**: Xie, S. *et al.* Key evidence of the role of desertification in protecting the underlying permafrost in the Qinghai–Tibet Plateau. *Sci. Rep.* **5**, 15152; doi: 10.1038/srep15152 (2015).

## Figures and Tables

**Figure 1:**
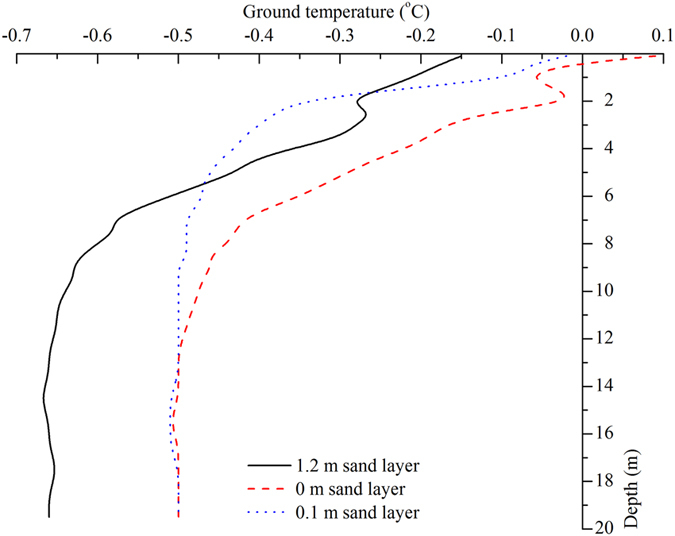
Temperature variation in the permafrost underneath the sand layer at the Honglianghe River of the Qinghai–Tibet Plateau (2011).

**Table 1:** Radiation observations for the sand layer and the surface without sand at the Honglianghe River of the Qinghai–Tibet Plateau (September 2012 to August 2013).

	Radiation	Sand layer	Without sand	Sand/no sand × 100%
Downward shortwave radiation	Annual average radiant flux density (W·m^−2^)	221.27	222.77	99.33
	Yearly total values (MJ)	6987.60	7034.69	99.33
Upward shortwave radiation	Annual average radiant flux density (W·m^−2^)	59.84	50.34	118.87
	Yearly total values (MJ)	1889.06	1589.25	118.86
Downward longwave radiation	Annual average radiant flux density (W·m^−2^)	214.81	216.45	99.24
	Yearly total values (MJ)	6773.29	6824.92	99.24
Upward longwave radiation	Annual average radiant flux density (W·m^−2^)	315.32	316.79	99.54
	Yearly total values (MJ)	9947.25	9993.67	99.54
Net radiation	Annual average radiant flux density (W·m^−2^)	60.92	72.09	84.51
	Yearly total values (MJ)	1924.55	2276.71	84.53

**Table 2:** Granularity of the sand and soil samples at the Honglianghe River of the Qinghai–Tibet Plateau.

Samples	Granularity (%)						
	2.00–1.00 mm	1.00–0.50 mm	0.50–0.25 mm	0.25–0.10 mm	0.10–0.05 mm	0.05–0.005 mm	0.005–0.001 mm
Sand sample	0.2	3.3	36.3	59.0	1.2	0	0
Soil sample	2.1	3.9	10.2	46.8	10.3	18.7	8.0
